# Circular RNAs (circRNAs) in Health and Disease

**DOI:** 10.3390/genes8120353

**Published:** 2017-11-28

**Authors:** Shahnaz Haque, Lorna W. Harries

**Affiliations:** RNA-Mediated Mechanisms of Disease Group, Institute of Biomedical and Clinical Sciences, University of Exeter Medical School, Barrack Road, Exeter EX2 5DW, UK; sh683@exeter.ac.uk

**Keywords:** Circular RNAs, back-splicing, gene regulation, biomarkers, human disease

## Abstract

Splicing events do not always produce a linear transcript. Circular RNAs (circRNAs) are a class of RNA that are emerging as key new members of the gene regulatory milieu, which are produced by back-splicing events within genes. In circRNA formation, rather than being spliced in a linear fashion, exons can be circularised by use of the 3′ acceptor splice site of an upstream exon, leading to the formation of a circular RNA species. circRNAs have been demonstrated across species and have the potential to present genetic information in new orientations distinct from their parent transcript. The importance of these RNA players in gene regulation and normal cellular homeostasis is now beginning to be recognised. They have several potential modes of action, from serving as sponges for micro RNAs and RNA binding proteins, to acting as transcriptional regulators. In accordance with an important role in the normal biology of the cell, perturbations of circRNA expression are now being reported in association with disease. Furthermore, the inherent stability of circRNAs conferred by their circular structure and exonuclease resistance, and their expression in blood and other peripheral tissues in association with endosomes and microvesicles, renders them excellent candidates as disease biomarkers. In this review, we explore the state of knowledge on this exciting class of transcripts in regulating gene expression and discuss their emerging role in health and disease.

## 1. Introduction

Circular RNAs (circRNAs) are an emerging class of RNA species that are present in species as diverse as archaea, flies, and humans [1,2,3,4]. circRNAs in higher organisms are reported to be produced by back-splicing events and can be synthesized from all regions of the genome, deriving mostly from exons but, less commonly, from antisense, intergenic, intragenic, or intronic regions [5]. circRNAs are both spatially and temporally regulated and evidence is emerging that they may have importance in normal development of tissues or organs but also in disease pathogenesis. Most circRNAs have been reported in the brain [2,6,7,8]. They can be found in most cell sub-compartments, but the majority localize predominantly to the cytoplasm [9]. circRNAs are inherently stable by virtue of their closed covalent structure and exonuclease resistance and are thought to be stable in exosomes [2,5,10,11,12]. This observation opens up the interesting possibility that circRNAs, like micro RNAs (miRNAs), may have roles in paracrine signalling or have roles in cell-to-cell cross talk. 

## 2. circRNAs Are Formed from Back-Splicing Events of Linear Genes

**Figure 1:**
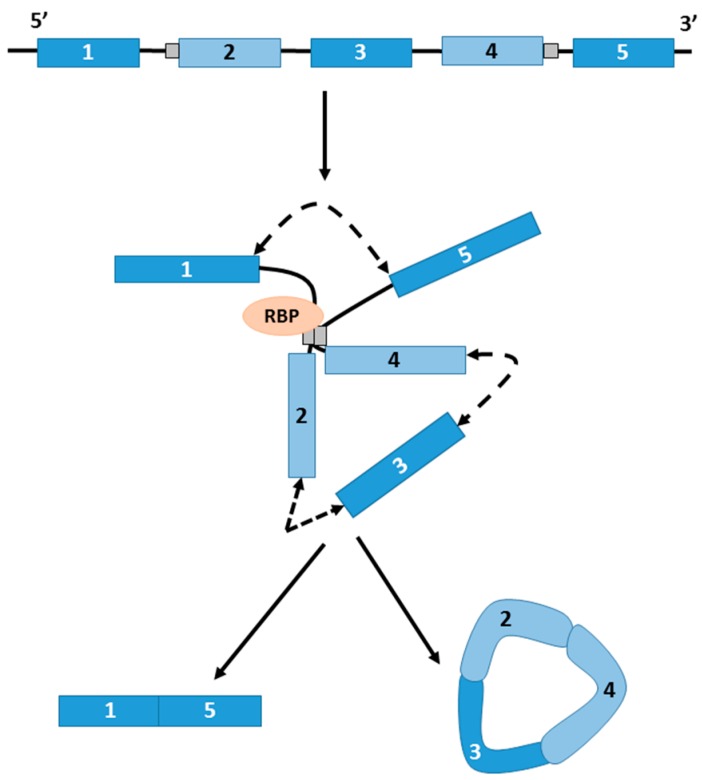
The biogenesis of circular RNA (circRNA). The linear primary transcript contains exons (blue boxes), introns (black lines), and possibly repetitive elements or sequence motifs (grey boxes). Circular exons are generated from back-splicing events between the splice donor site of a downstream exon and the splice acceptor site of an upstream exon. This can be mediated by specific sequence elements (grey boxes) or by interaction with RNA binding proteins (RBPs). Splicing events are indicated by dashed lines with double arrowheads. This may result in the production of a circular RNA and a linear RNA which lacks the circularised exons.

In conventional linear splicing, the spliceosome joins exons in a 5′ to 3′ configuration. In contrast, circRNAs arise when the 3′ ‘tail’ of a downstream exon of a gene is back-spliced to the 5′ ‘head’ of an earlier exon (which may include itself) leading to the circularization of exons in between (see Figure 1) [10]. These splicing decisions are, as in linear splicing, regulated by *trans*-acting splicing factors and *cis* sequence elements [13]. Several sequence features influencing circRNA formation have been described. Firstly, intron length has been reported to play a part; introns flanking back-spliced sites tend to be comparatively longer than those flanking non-circularised exons [4]. This may be because larger introns may form more RNA–RNA interactions, facilitating circularization of embedded exons; the double-stranded RNA-editing enzyme ADAR1, which is capable of melting stem structures within these RNA–RNA interactions, is associated with suppression of circRNA expression in *Caenorhabditis elegans* [14]. Secondly, exon length may also be a factor; exons of single-exon circRNAs are on average 3-fold longer compared with those of non-circularised exons; longer exons may be sterically preferentially favoured for 3′–5′ splicing at canonical splice sites [2,15,16]. Thirdly, RNAs that are hyper-edited are enriched for circRNA sequences [14]. Finally, sequence content may also be important. Repetitive sequences are known to promote back splicing; back-spliced exons that form circRNAs are frequently enriched in paired ALU tandem repeats that have been shown to promote circularization [2]. Miniature introns with as few as 30 to 40-nt inverted repeats are also sufficient to promote circularization [17]. 

circRNA formation may also be dependent on the specific binding of regulatory proteins. RNA binding proteins such as Quaking (QKI) and Muscleblind (MBL/MBNL1) have been described to bind to introns flanking back-spliced sites and may drive circularization [18,19]. The *MBL* gene itself encodes a circular form which regulates the expression of its linear transcript and modulation of MBL levels strongly affects circMBL expression [18]. circRNA formation has also been shown to depend on the rate of transcription of their parent genes. circRNA producing genes are generally longer and exhibit faster transcription than genes that do not produce circRNAs, and artificially slowing the rate of transcription with mutant RNA polymerases results in lower levels of circRNA biogenesis [20]. 

Intronic circRNAs (ciRNAs) can also be generated form lariat introns. ciRNAs are devoid of linear fragments spanning the 3′ end of the intron to the branch point, but are produced by a 2′,5′-phosphodiester bond arising from canonical linear splicing [21]. A 7-nt GU-rich element occurring close to a 5′ splice site and with an 11-nt C-rich motif around the branch point within intronic sequences has been reported to be important for formation of ciRNAs [21].

## 3. circRNA Online Resources 

Over the past few years, at least eleven circRNA detection software systems have been developed. These tools can recognize circRNA sequences from RNA-Seq data based on two different strategies. One approach is the candidate-based approach, also known as the pseudo-reference based approach. KNIFE, NCLScan, and PTESFinder all abide by this approach where putative circRNA sequences need to be provided with information for gene annotation. Although KNIFE can directly retrieve back-spliced junctions prior to gene annotations, the other two software systems generate putative circRNA sequences post-alignment with the genome or transcriptome [22,23,24]. 

circRNA_finder, CIRCexplorer, DCC, MapSplice, Segemehl, find-circ, and UROBORUS detect circRNA sequences based on the second approach for identification of circRNA sequences which is the fragmented-base or segmented read approach. In this approach, the software detects back-spliced sites based on reads mapped to alignment of multiple split reads against the genome. While find-circ and UROBORUS detect back-spliced sequences using the first and last 20 bp after mapping the sequences against the genomes, the rest of the detection tools generate splice alignment algorithms to identify back-spliced junctions [22,23,24]. CIRI is a distinct detection tool in that it extracts information from local alignment with Burrows-Wheeler Aligner-MEM (BWA-MEM) and detects paired chiastic clipping signals from mapped reads (arXiv:1303.3997). Comparisons of the different circRNA detection algorithms have now been made, which add information on the relative strengths and caveats of these different approaches [25]. Searchable repositories for circRNA sequences such as circBase are also now emerging [26], which should prove useful to researchers interested in these RNA species in the future. 

## 4. Molecular Mechanisms of Gene Regulation by circRNAs

**Figure 2:**
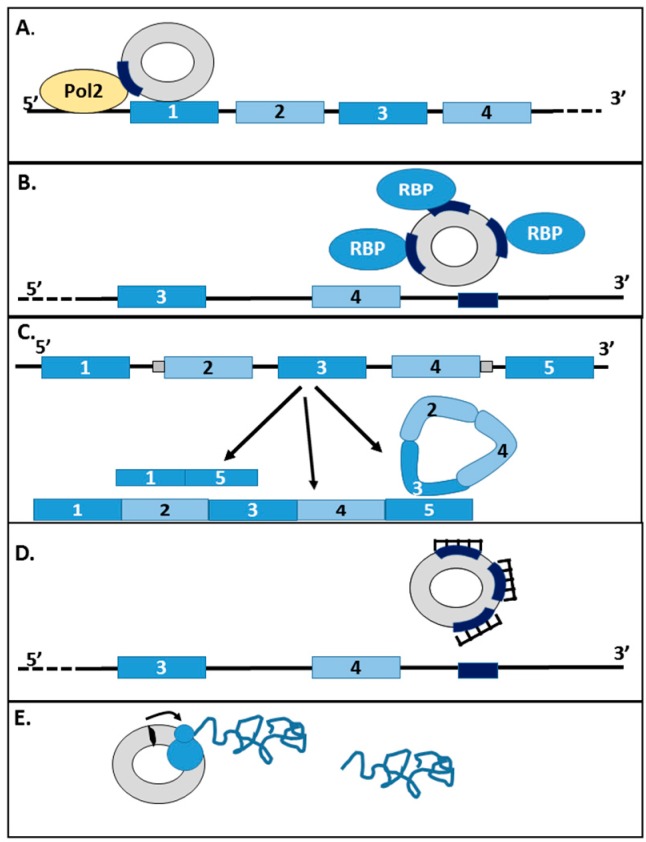
Proposed roles of circRNA in the regulation of transcription and translation. circRNAs may regulate genes at several levels. (**A**) Firstly, nuclear circRNAs can interact with promoter regions of target genes and interact with RNA polymerase II (Pol2) to repress or enhance transcription; (**B**) Secondly, circRNAs can sequester RBPs that regulate mRNA processing and, thus, alter the splicing patterns of the genes in question, or moderate mRNA stability. RBP binding sites are given by dark blue boxes; (**C**) Thirdly, the biogenesis of circular RNAs may results in the production of a linear RNA lacking the circularised exons. The formation of circRNAs can thus reduce the amount of linear transcript produced; (**D**) circRNAs can act as micro RNA (miRNA) sponges, sequestering them away from their binding sites in target genes, which are given by dark blue boxes; (**E**) Circular RNAs can also be translated. The initiation codon is given by a black oval, and the translating ribosome and nascent polypeptide are indicated.

circRNAs have been proposed to act through several mechanisms, including miRNA sponges, modifiers of transcription or translation, and as splicing modifiers (Figure 2).

### 4.1. circRNAs as miRNA Sponges

circRNA can bind specific miRNAs or groups of miRNAs, sequestering them and suppressing their function [27], in a phenomenon termed the competitive endogenous RNA hypothesis [28]. circRNA *CDR1as* has been documented to contain up to 74 binding sites for the miRNA miR-7, and also binds Argonaute (AGO) proteins of the RNA-induced silencing complex (RISC) that regulate miRNA action [29]. There is also some suggestion that the relationships between circRNAs and miRNAs may be partly autoregulatory; *CDR1as* also binds miR-671, which induces AGO-mediated cleavage of *CDR1as* itself, which could act to release miR-7 [30]. However, circRNAs containing multiple binding sites for single miRNAs may be the exception rather than the rule since most circRNAs identified to date do not contain enrichment of binding sites for specific miRNAs [31]. Emerging evidence suggests that circRNAs may act by sequestration of modules of coordinately regulated miRNAs; the circRNA *circHIPK3* contains binding sites for nine miRNAs with growth-suppressive properties [32]. The presence of multiple binding sites may not be a prerequisite for efficient miRNA regulation, however, since *circHIPK3* contains only two binding sites for miR-124, yet retains the ability to regulate this miRNA [32]. Most of the research investigating the role of the circRNA–miRNA interaction has been performed through correlation of the levels of miRNA and circRNA expression in vitro. Evidence for circRNAs acting as miRNA sponges can also be seen in data arising from the *CDR1as* knockout mouse. Levels of both miR-7 and miR-671 were seen to be lower in knockout animals, and these changes were also correlated with defects in synaptic transmission [33]. It is likely that a circRNA with multiple binding sites will affect the expression of a larger number of miRNA targets. However, experimental validation of the minimal number of miRNA binding sites for a candidate circRNA to be functional is still required for many circRNAs. An arena to explore is whether a single miRNA binding site would be sufficient for efficient circRNA:miRNA sponging interactions. It would be also interesting to know what levels of circRNA expression is required for the optimal miRNA sequestration ability of these entities. The interaction between circRNAs and miRNAs may also go beyond their role in miRNA sequestration; they may also be important for the storage, sorting, and localization of miRNAs, adding an additional level of regulation to miRNA-controlled regulation of target genes [27,34]. 

### 4.2. circRNAs as Transcriptional and Translational Regulators 

A specific category of circRNAs, nuclear exon-intron circRNAs (EIciRNAs) can also interact with the transcription machinery. These variant circRNAs, which retain some intronic sequence from their linear gene, can interact with the U1 component of the spliceosomal machinery, recruiting RNA polymerase II to the promoter region of genes and enhancing expression of its target genes [35]. circRNAs can also modulate the expression of the cognate transcript if the circularisation event includes the translation initiation codon of its native gene. This may cause their cognate linear mRNAs arising from the same gene to escape translation, thus regulating protein expression as in the case of *circHIPK3*, *circDMD,* and *circFMN* [2,36,37,38]. Recently, *circPABPN1* has been reported to supress binding of *PABPN1* mRNA to HuR. *PABPN1* translation is positively associated with HuR and the interaction of *circPABPN1* interaction with HuR reduces the translational efficiency of *PABPN1* transcripts. Thus, circRNAs like *circPABPN1* can act as competitors with their cognate mRNA for RBPs and can also modulate the rate of translation of target mRNAs [39].

### 4.3. circRNAs as Competitors of Linear Splicing

All isoforms produced from a given gene arise from a common pre-mRNA. It follows, therefore, that the production of a circRNA may have consequences for the abundance of the remaining transcripts encoded by that gene. An example of this lies in the Muscleblind (*MBL*) gene. *MBL* contains sequences that form a circRNA transcript that contains binding sites for MBL itself. Production of the circMBL therefore forms an autoregulatory loop that regulates the production of the linear transcript in favour of the circular form [18]. In *Arabidopsis*, a circRNA derived from the *SEPALLATA3* gene has been shown to interact with its cognate DNA, forming a R-loop and causing a pause in transcription and also affecting recruitment of splicing factors to the nascent transcript and affecting alternative splicing through exon-skipping [40]. 

### 4.4. circRNAs as Sponges (RBPs)

In addition to their role as miRNA sponges, circRNAs can also act as sponges for other entities such as RBPs that can regulate gene expression. RBPs, like miRNAs, bind specific sequences within their target genes and control all stages within the lifecycle of an mRNA from splicing and nuclear export to stability and subcellular localisation [36]. circRNAs interacting with RNA binding protein components of the gene regulatory machinery such as HuR have been reported [39]. 

## 5. Translation of circRNAs 

Recent in vitro studies have shown that circRNAs have potential to encode proteins. Ribosome footprinting studies in vivo in Drosophila clearly demonstrate that circRNAs are associated with translating polysomes [41]. Accordingly, some circRNA-derived proteins have been identified. For example, circ-FBXW7 has been shown to encode a novel protein in human U251 and U373 cell lines [42]. The inclusion of N6-meythyl adenosine residues is sufficient to promote the initiation of translation of circRNAs in the human cell lines in the presence of initiation factor eIF4G2 and YTHDF3 [43]. Methyltransferase METTL3/14 also accelerates the initiation of translation of this circRNA [43]. Computational analysis of high-throughput sequencing data revealed that the human transcriptome commonly harbours many circRNAs with coding potential. Smaller circRNAs with relatively fewer exons but longer open reading frames (ORFs) have been reported to be associated with polysomes [43]. For example, circ-ZNF609 has been demonstrated to encode a protein in a splicing-dependent but cap-independent manner in human and murine myoblasts. In vitro analysis of circ-ZNF609 (which contains two start codons) revealed that the circRNA could generate two protein isoforms corresponding to the two ORFs [6]. 

## 6. The Roles of circRNAs in Normal Homeostasis

circRNAs are emerging as important regulators of many cellular processes, such as embryonic development, control of cell cycle, cellular senescence, cell signalling, and response to cellular stress.

### 6.1. circRNAs in Embryonic Development

Approximately 10.4% of human circRNAs and 34.3% of mouse circRNAs are expressed in a tissue specific or age-specific manner, which indicates their potential role in tissue development or differentiation [44]. The difference in abundance of circRNAs between human and mouse is interesting and may represent a real species difference, but may also be a reflection of limited power due to smaller sample numbers of ex vivo human studies because of the inherent difficulties in procuring non-accessible tissues. The albumin (*ALB*) gene generates up to 160 circRNA species, of which 95 are specific to adult liver and only 33 are expressed in developing foetal liver [44]. circRNAs are enriched in the brain and appear to have particular importance in brain development of this organism. *CiRS-7* has been shown to be highly expressed in the cerebellum at embryonic stage E115, but reduced in the cerebral cortex at E60 in embryonic pigs [7]. Embryonic, early postnatal, postnatal, and late postnatal hippocampus from brain samples from embryonic mice also had increased levels of *circDlgap1*, *circMyst4*, *circKlhl2,* and *circAagab*. Expression was localized to the punctate in the dendrites, and abundance fluctuated depending on the stage of synaptogenesis, suggesting that these circRNAs might be involved in synaptic function during developmental stages [45]. *CDR1as* may have important roles in development, since transgenic expression of this circRNA in zebrafish embryos produces fry with smaller midbrain size, which resembles the phenotype of miR-7 knockdown [29]. The *SRY* gene is located in the sex-determining region of the Y chromosome. Mutations in the coding regions of *SRY* can lead to change in sex [46,47]. Mouse *circSRY* has multiple miR-138 binding sites suggesting a potential role for this circRNA in the regulation of sex determination in mice [29,30]. 

### 6.2. circRNAs in Metabolism

High *CDR1as* expression has been shown to have effects on beta-cell function through its regulation of miR-7 in pancreatic beta cells. miR-7 targets include the protein kinase C beta (*PKCB*) gene involved in cellular signalling, the profilin 2 (*PFN2*), and phosphatase and actin regulator 1 (*PHACTR1*) genes involved in cytoskeletal organisation and the gene encoding the transcription factor paired box 6 (*PAX6*). This may have profound implications for beta cell function; up-regulation of murine *Pax6* expression through the sponging action of *CDR1as* on miR-7 has been shown to lead to increased insulin secretion in mouse islets [48]. miR-7 is abundant in adult islets and may prevent β cell proliferation by inactivating the mTOR pathway [49]. 

### 6.3. circRNAs in Regulation of Cell Cycle

circRNAs have been found to be involved in the progression of the cell cycle. *circFOXO3* has been shown to engage with p21 and cyclin-dependent kinase 2 (CDK2) proteins to form a ternary structure. The complex formed prevents CDK2 from interacting with cyclin E and p27, which eventually blocks transition from G1 to S phase and cell cycle progression. Similarly, p21, like CDK2, is unavailable for interaction with cyclin A, which leads to cell cycle arrest at G1 phase [50]. Depletion of *circHIPK3* in HEK293T cells has also been shown to supress cell proliferation and is indicative of a role in cellular growth [32]. 

### 6.4. circRNAs in Regulation of Cellular Stress

It is vital for living organisms to maintain homeostasis at the cellular level to sustain viability. circRNAs have been reported to be involved with cellular homeostasis both positively and negatively, by regulating aspects of cellular growth, apoptosis, immune-response, and resistance to therapeutics. *CDR1as* has been shown to have potential roles in the regulation p21-activated kinase 1 (PAK1), promoting DNA repair and preventing apoptosis [51]. Mouse embryonic fibroblasts exposed to reactive oxygen species demonstrate upregulation of *circFOXO3*, promoting senescence via reducing nuclear translocation of ID-1, E2F1, and HIF1α and altering the mitochondrial localization of focal adhesion kinase (FAK), all of which are involved in cellular survival [50]. A circRNA derived from the *DENND4C* gene has also been reported to have roles in adaptation to hypoxic conditions in breast cancer cells [52]. Overexpression of a circRNA produced from the locus encoding the lncRNA *ANRIL* has been shown to induce prolonged nucleolar stress in cultured human cells [53]. circRNAs have also been reported to have roles in macrophage activation and antimicrobial response via positive regulation of the intracellular adhesion molecule 1 (*ICAM1*) gene in the Toll-like receptor 4 (TLR4) pathway [54]. 

## 7. circRNAs in Disease

In accordance with a pivotal role in gene regulation, perturbation of circRNA expression is beginning to be reported in association with disease. Altered circRNA expression has been reported in several diseases like cancer, heart disease, neurological disorders, diabetes and atherosclerosis, although the precise mechanisms by which they operate are yet to be disclosed. 

### 7.1. circRNAs in Cancer 

In accordance with their known role in modulating cell cycles, proliferation and cellular senescence as demonstrated in vitro studies, circRNAs have been implicated in cancer. circRNA hsa_circ_001569 (circABCC) may play a role in modulating gene expression in colorectal cancer by virtue of its action on miR-145. miR-145 is a negative regulator of target genes such as *E2F5, BAG4,* and *FMNL2* which are known to be involved in the suppression of proliferation [55]. Similarly, *CDR1as* has also been implicated in several cancer subtypes including hepatocellular carcinoma (HCC). *CDR1as* expression is known to be correlated with hepatic microvascular invasion in HCC tissue [56] and hsa_circ_0000520 (circRPPH1), hsa_circ_0005075 (circEIF4G3), hsa_circ_0066444 (circADAMTS9), and hsa_circ_0001649 (circSRPRH) have all been shown to be expressed at different levels in HCC compared to adjacent normal liver tissues [57,58]. hsa_circ_0005075 (circEIF4G3) correlated with tumour size while hsa_circ_0001649 (circSRPRH) was downregulated and also correlated with tumour size in addition to the prevalence of tumour embolus and could be involved in tumorigenesis and metastasis of HCC [57,58]. circRNA *circZKSCAN1* has also been shown to be lower in tumours and correlated with tumour size in HCC [59].

Large-scale dysregulation of circRNA expression has been noted in bladder carcinoma, where microarray analysis revealed that 469 circRNA were differentially expressed, 285 showing increased expression in bladder cancer compared with 184 showing downregulation. Of these, *circTCF25* regulates miRNAs miR-103a-3p/miR-107, with *circTCF25* upregulation being associated with increased levels of 13 targets associated with cell proliferation, migration, and invasion in bladder cancer [60]. Similarly, increased expression of circRNA *circMYLK* in bladder cancer leads to overexpression of *DNMT3B*, *VEGFA,* and *ITGB1* genes, which are involved in promotion of proliferation and are molecular targets of miRNA-29a-3p [61]. circRNA *circHIAT1* has also been reported to respond to signalling through the androgen receptor to promote tumour migration and invasion in clear cell renal cell carcinoma by enhancement of *CDC42* expression through regulation of miR-195-5p/29a-3p and miR-29c-3p target genes [62]. Recently, fusion-circRNAs (f-circRNA) have been shown to accelerate proliferation rate and instigate cellular transformation. Furthermore, f-circRNAs f-circM9 and f-circPR have been shown to confer resistance to therapeutics due to protective effects of drug-induced apoptosis in cancer cells in vitro via a MAPK/AKT dependent signalling pathway [63]. 

### 7.2. circRNAs in Neurological Disease 

*CDR1as* has been associated with neurodegenerative conditions such as Alzheimer’s disease (AD). In patients with moderate to advanced stages of sporadic AD, *CDR1as* expression has been reported to be reduced, which may lead to elevated miR-7 expression and consequent downregulation of miR-7 dependent mRNAs. One target, ubiquitin protein ligase A, is responsible for the clearance of amyloid peptides in AD and other degenerative disorders [64]. Although most studies with circRNAs have been conducted in vitro, recently, Piwecka et al. generated a knockout murine model for Cdr1as. The brains of these transgenic mice had upregulated expression of miR-7 genes, such as *c-Fos*. Cdr1as knockout mice demonstrate impaired synaptic transmission and information processing defects [33]. circRNAs also have potential roles in memory; *circPAIP2* has been suggested to upregulate memory-related gene *PAIP2* through the poly A binding protein (PABP)-associated pathway [65]. A role for circRNAs in major depressive disorder is also suggested by the observation that a significant change of hsa_circRNA_103636 expression was noted in patients after eight weeks on antidepressant therapies [66]. 

### 7.3. circRNAs in Osteoarthritis

Osteoarthritis (OA) occurs because of degenerative changes in the joint cartilage. Seventy-one circRNAs were seen to be differentially expressed in the cartilage of patients with OA compared with those of non-OA controls. The circRNA *circ-CER* appears to be of particular importance. *circ-CER* expression was shown to increase with increased expression of pro-inflammatory signalling molecules such as interleukin (IL)-1 and tumour necrosis factor (TNF)α. Suppression of *circ-CER* resulted in reduced matrix metalloproteinase-13 (*MMP13*) expression and remodelling of the extracellular matrix (ECM). The authors suggest that this observation arises from a sponging effect of *circ-CER* on miR-136, which is known to target the *MMP13* gene [67]. 

### 7.4. circRNAs in Cardiovascular Disease 

The *CDKN2A/CDKN2B* locus expresses an alternatively spliced non-coding transcript, *ANRIL*, which encodes a circular form in addition to its linear form. *circANRIL* has been reported to regulate the pescadillo homologue 1 (*PES1*) gene transcript, which is involved in pre-rRNA processing and ribosome biogenesis. Sequestration of this essential factor and suppression of these key processes in vascular smooth muscle cells and macrophages was shown to cause nucleolar stress and p53 activation, resulting in apoptosis and features of atherosclerosis [68]. Some circRNAs have been shown to be protective in heart function; the circRNA heart-related circRNA *HRCR* has been implicated in protection from cardiac hypertrophy and heart failure, by virtue of its binding of the inflammatory onco-miR miR-223 [69,70]. Some RNA inding Motif Protein 20 (RBM20)-dependent circRNAs have also been reported to be differentially regulated in dilated cardiomyopathy [71]. Similarly, circRNA *MFACR* has been shown to sponge miR-652-3p in the cytoplasm, promoting mitochondrial fission and cardiomyocyte cell death by enhancing translation of *MTP18* in animal models [72]. Similarly, high levels of circRNA_000203 and circRNA_010567 have been reported in cardiomyocytes from diabetic mice treated with angiotensin II. These circRNAs are thought to downregulate miR-26b-5p, miR-141, and miR-141, thereby upregulating the *TGFB1* gene. This leads to suppression of fibrosis-associated protein resection in collagen 1 (Col I), collagen 3 (Col III), and α-smooth muscle actin (α-SMA), promoting fibrosis in the myocardium [73,74]. 

### 7.5. circRNAs in Type 2 Diabetes 

In addition to the role of *CDR1as* in regulation of insulin secretion through modulation of PAX6, recent studies have suggested that circRNAs may have utility as biomarkers of diabetes. Four hundred eighty-nine circRNAs were found to be differentially expressed in the peripheral blood of patients with type 2 diabetes, and furthermore, circRNA hsa_circ_0054633 was found to be capable of predicting pre-diabetes with an area under the curve (AUC) of 0.84 (*p* ≤ 0.001) [75]. In other studies, several circRNAs have been shown to be differentially expressed in serum of patients with diabetic retinopathy compared to that of both controls and diabetes patients without retinopathy. Of these, hsa_circRNA_100750 is derived from stromal interaction molecule 1 which is upregulated in diabetic patients. hsa_circRNA_104387 is known to sequester miR-29a which prevents the loss of renal function in diabetic patients. hsa_circRNA_103410 is known as a regulator of miR-126, which is known to inhibit *VEGF* and *MMP9* expression. Thus, hsa_circRNA_103410 could promote endothelial injury in the retina, while hsa_circRNA_100192 could, by sequestering miR-146, promote necrosis factor (NF)-κB activation, adenosine deaminase-2 expression, and inflammatory responses as is observed in vitro [76]. These observations raise the exciting possibility that circRNAs expressed in accessible tissues may be useful markers of disease in inaccessible organs such as pancreas. *circHIPK3* has been found to be dysregulated in diabetic retinas, which may contribute to elevated levels of *VEGFC*, *FZD4*, and *WNT2* expression by virtue of its effects on miR-30a-3p [77]. 

### 7.6. circRNAs and Pre-Eclampsia

A study has implicated circRNAs in the development of preeclampsia. Qian et al. identified 143 up-regulated and 158 down-regulated circRNAs in placental tissues from women with pre-eclampsia, which included upregulated hsa_circRNA_100782 (circHIPK3), hsa_circRNA_102682 (circCRIM1), and hsa_circRNA_104820 (circFAM120A) circRNAs [78]. Altered circ_101222 expression before 20 weeks of pregnancy was seen to correlate with higher levels of endoglin, a component of the transforming growth factor beta (TGFβ) signalling pathway, which is associated with pre-eclampsia, than women who did not have pre-eclampsia. circRNA circ_101222, in combination with endoglin levels, may therefore have the potential to predict pre-eclampsia very early on in pregnancies [79].

### 7.7. circRNAs and Infection

Microbial lipopolysaccharide induces the activation of TLR pathways leading to activation of NF-κB and modulation of genes which are key to antimicrobial defences and adaptive immunity. *circRasGEF1B* has been suggested to modulate the expression of ICAM-1 as part of the lipopolysaccharide response. Knock down of this circRNA in vitro leads to 27%–39% reduction in ICAM-1epression in vitro [54]. In mouse macrophage cells, activation of TLR4, TLR9, TLR3, TLR2, and TLR1 receptors all regulate the expression of *circRasGEF1B* [54]. Genetically modifying *circRasGEF1B* expression was shown to reduce *ICAM1* expression levels, which under normal conditions would promote binding of leukocytes to endothelium cells and their transmigration into target tissues [54]. Thus, deficiency of *circRasGEF1B* may prevent migration of leukocyte cells to inflammatory sites and interfere with the healing process, and in cancer cells may additionally affect the activation of cytotoxic T-lymphocytes needed for driving release of cytolytic granules into tumour cells [54]. 

A potential role for circRNA in response to viral infection has been reported. HeLa cells transfected with circRNA demonstrated induced expression of 84 innate immunity-related genes, such as *RIGI* and *OASl*, which were upregulated by as much as 500-fold and 200-fold, respectively [80]. Concurrent with these changes was a 10-fold decrease in infection rate against Venezuelan equine encephalitis virus, which was shared by nearby non-transfected cells, indicating some paracrine action [80]. circRNAs can also act as competitors with viral mitochondrial RNA (mtRNA) for binding to RNA-binding domain containing immune factor NF90 and its isoform NF110. These factors can promote circularization by stabilizing the binding of intronic RNA pairs in the nucleus. Viral infection results in transportation of these factors from the nucleus to the cytoplasm resulting in decreased circRNA expression in infected cells. This acts to render the circRNA available for binding to viral mRNA and prevents viral infection of the host cell [81]. 

### 7.8. circRNAs in Ageing and Cellular Senescence

circRNAs are known to accumulate in ageing brains [82]. This may be partly due to the endonuclease-resistant nature of circRNA molecules, but at least two circRNAs have been previously described to have a role in ageing or cellular senescence. circRNA *circPVT1* has been demonstrated to suppress cellular senescence by sequestration of miRNA let-7, which lifts its inhibitory action on its target genes *IGF2BP1*, *KRAS,* and *HMGA2,* which act to promote cell proliferation [83]. Conversely, the *circFOXO3* circRNA was found to promote senescence in the heart muscle of aged mice and humans through its action on the *ID1*, *E2F1*, *FAK,* and *HIF1A* target genes [9]. This circRNA is also known to silence cell proliferation through its regulation of the cell division kinase 2 (*CDK2*) gene and the cyclin dependent kinase inhibitor p21 [9].

## 8. circRNAs as Diagnostic and Prognostic Markers 

Because circRNAs are highly nuclease-resistant, they are more stable than linear transcripts and may be released into the extracellular space via the exosomes [12,84]. Half-lives of circRNAs can vary significantly, but can be as long as 50 h. On average, their half-lives are around 2.5-fold longer than the median half-lives of their linear counterparts [85]. A substantial number of circRNAs are expressed in blood at comparatively higher levels than their linear mRNAs, thus making circRNAs attractive tools for diagnostics to trace the mechanism of coded genes otherwise inaccessible by the canonical RNA pathway-dependent assays [86]. A selection of circRNAs reported as potential biomarkers for different diseases are summarized in Table 1. Over 400 circRNAs have been detected to be present in cell-free saliva and could potentially be used for non-invasive diagnostics approach [87]. circRNAs have been suggested as potential biomarkers for several types of cancer. In murine models, tumour-derived exosomal circRNAs in the serum correlate with tumour mass [88], and thus may be promising biomarkers for cancer detection. circRNA hsa_circ_0001649 (circSHPRH) has shown some utility as a biomarker for hepatocellular carcinoma, is downregulated with tumour status, and is associated with the occurrence of the tumour embolus as well as the size of the tumour [57]. hsa_circ_002059 (circKIAA0907) is downregulated in gastric cancer and is associated with grade and distal metastasis and could, therefore, be used as a prognostic marker [89]. Another circRNA, hsa_circ_0000190 (circDYRK1A), is downregulated in plasma samples of patient with gastric cancer, where its expression levels correlated with tumour diameter, lymphatic metastasis, and distal metastasis [90]. Similarly, hsa_circ_0001895 (circPRRC2B) was also shown to be downregulated in gastric cancer tissues and correlated with cell differentiation and Borrmann type [91]. In hepatitis B infected hepatocellular carcinoma (HCC) patients, circRNA_100338 (circSNX27) has been shown to regulate levels of miR-141-3p, and its expression correlated with low cumulative survival rate and metastatic progression [92]. 

## 9. Conclusions 

circRNAs are a class of non-coding RNAs which appear to regulate the expression of genes by a variety of mechanisms and might also have the potential of encoding proteins, the mechanisms of which are not yet completely understood. Despite this, circRNAs are emerging as potentially important regulators of cellular physiology and as potential biomarkers of disease onset or progression. The state of the current knowledge of circRNA biology is as yet at a very early stage, and further research is urgently required to completely understand their function and potential. Despite this, it is likely that these novel circRNAs will emerge as important players in gene regulation in the future. The origin of circRNAs from throughout the genome raises the possibility of co-ordinated regulation of modules of genes in a cell, tissue, and developmental stage specific pattern, adding another level of regulation to the already complex field of non-coding RNA (ncRNA) regulation of gene expression. In future years, circRNAs could be exploited as therapeutics. Over-expression of specific circRNA constructs could act to modulate cell behaviour and physiology by sponging oncogenic miRNAs such as miR-21 and miR-221 in cancer cells [108]. Antisense approaches such as morpholino technologies to influence splicing patterns are already in development for diseases such as Duchenne muscular dystrophy [109] and could in theory be developed to target exons that may be circularized to bring about higher expression of beneficial circRNAs once their modes of action have been elucidated.

## Figures and Tables

**Table 1:** Examples of circRNA and their potential role in disease.

circRNA	Pathologic Condition	Possible Mode of Function	Potential Application
hsa_circRNA_062557, hsa_circRNA_067130, hsa_circRNA_067209, hsa_circRNA_100914, hsa_circRNA_089761, hsa_circRNA_089763	moyamoya disease	May sequester miRNAs associated with RNF213 and BRCA1/BRCA2-containing complex subunit 3	Potential biomarker expressed in blood [93]
CDR1as	hepatocellular carcinoma	May be a sponge for miR-7	Biomarker with the ability to predict hepatic microvascular invasion; expressed in hepatocellular carcinoma tissues [56]
hsa_circ_0001017, hsa_circ_0061276	gastric cancer		Prognostic, with the ability to predict disease-free survival; expressed in plasma [94]
hsa_circ_0089378, hsa_circ_0083357, hsa_circ_0082824, hsa_circ_0068942, hsa_circ_0057576, hsa_circ_0054537, hsa_circ_0051172, hsa_circ_0032970, hsa_circ_0006323	coronary artery disease	May promote expression of transient receptor potential cation channel subfamily M member 3 by inhibiting hsa-miR-130a-3p	Potential biomarker expressed in plasma [95]
GSDMB circRNA	multiple sclerosis		Potential biomarker expressed in peripheral blood mononuclear cells [96]
hsa_circRNA_105055, hsa_circRNA_086376, hsa_circRNA_102761	colorectal cancer	May act as sponge for miR-7 regulating target genes *PRKCB*, *EPHA3*, *BRCA1,* and *ABCC1*; potential role in lung metastasis	Potential biomarker [97]
hsa_circ_0092285, hsa_circ_0058794, hsa_circ_0088088, hsa_circ_0038644	rheumatoid arthritis	May be involved in response to oxidative stress; endocytic traffic in actin cytoskeleton; could promote lipid breakdown and increase free fatty acid levels; could alter lipopolysacccharide (LPS) immune response	Potential biomarker expressed in peripheral blood mononuclear cells [98]
hsa_circRNA_101308, hsa_circRNA_104423, hsa_circRNA_104916, hsa_circRNA_100269	gastric cancer		May predict the early recurrence of stage III gastric cancer after radical surgery; expressed in tumour tissues [99]
circPVT1	gastric cancer	May act as sponge for miR-125 family; may promote cell proliferation	Potential prognostic marker with the ability to predict overall survival and disease-free survival; expressed in gastric cancer tissues [100]
circRNA_104871, circRNA_003524, circRNA_101873, circRNA_103047	rheumatoid arthritis		Potential biomarker expressed in peripheral blood mononuclear cells [101]
hsa_circ_0058246	gastric cancer		Potential prognostic marker with the ability to predict clinical outcome; expressed in tumour tissues [102]
circ-ITCH	hepatocellular carcinoma	May inhibit Wnt/β-Catenin pathway	Potential prognostic marker with the ability to predict survival; expressed in hepatocellular carcinoma tissues [103]
hsa-circ-0005870	hypertension	May act as sponge for miRNAs, hsa-miR-6807-3p, hsa-miR-5095, hsa-miR-1273g-3p, hsa-miR-5096, and hsa-miR-619-5p, possibly affecting transforming growth factor beta (TGF-beta) pathway important in hypertension	Potential biomarker expressed in plasma [104]
hsa_circ_0124644,	coronary artery disease		Potential diagnostic biomarker; expressed in blood [105]
circR-284	carotid disease and ischemic stroke	May act as an inhibitor of miR-221/miR-222	Potential diagnostic biomarker:expression demonstrated in serum [106]
circ_0005402, circ_0035560	multiple sclerosis		Potential biomarker; expressed in leucocytes [107]
circZKSCAN1	hepatocellular carcinoma	May modulate expression of apoptotic genes *RAC2*, *EFNA3*, and caspase 3 and cell proliferation related genes *TGFB1*, *ITGB4, CXCR4*, *BIRC5,* and *CCND1*; may modulate promoted cell proliferation, migration, and invasion in vitro	Expressed in tumour tissues [59]
circ_101222	pre-eclampsia		Potential biomarker; expressed in blood [79]
hsa_circ_0054633	diabetes		Potential biomarker with the ability to predict pre-diabetes and type 2 diabetic status; expressed in blood [75]
